# Electric Current Dependent Fracture in GaN Piezoelectric Semiconductor Ceramics

**DOI:** 10.3390/ma11102000

**Published:** 2018-10-16

**Authors:** Guoshuai Qin, Chunsheng Lu, Xin Zhang, Minghao Zhao

**Affiliations:** 1 School of Mechanics and Engineering Science, Zhengzhou University, Zhengzhou 450001, Henan, China; gsqin0404@163.com (G.Q.); 18337161838@163.com (X.Z.); 2 School of Civil and Mechanical Engineering, Curtin University, Perth, WA 6845, Australia; c.lu@curtin.edu.au; 3 School of Mechanical Engineering, Zhengzhou University, Zhengzhou 450001, Henan, China; 4 Henan Key Engineering Laboratory for Anti-Fatigue Manufacturing Technology, Zhengzhou University, Zhengzhou 450001, Henan, China

**Keywords:** GaN piezoelectric semiconductor ceramics, mechanical-electrical loading, intensity factor, fracture criterion

## Abstract

In this paper, the fracture behavior of GaN piezoelectric semiconductor ceramics was investigated under combined mechanical and electric loading by using three-point bending tests and numerical analysis. The experimental results demonstrate that, in contrast to traditional insulating piezoelectric ceramics, electric current is a key factor in affecting the fracture characteristics of GaN ceramics. The stress, electric displacement, and electric current intensity factors were numerically calculated and then a set of empirical formulae was obtained. By fitting the experimental data, a fracture criterion under combined mechanical and electrical loading was obtained in the form of an ellipsoid function of intensity factors. Such a fracture criterion can be extended to predict the failure behavior of other piezoelectric semiconductors or devices with a crack, which are useful in their reliability design and applications.

## 1. Introduction

Piezoelectric semiconductor ceramics (PSCs) are semiconducting ceramic materials that have piezoelectric properties [1,2,3]. Since the piezoelectric effect was discovered in ZnO and CdS semiconductors [4], the mechanical properties of PSCs have been intensively studied. In recent years, while considering the special interaction between the mechanical force and charge carrier of PSCs, numerous new PSC-based electromechanical devices have appeared, including ultrasonic transducers [5,6,7,8], sensors, and piezoelectric charge-coupled devices [9,10,11].

However, PSCs, as a typical kind of brittle ceramics, are highly sensitive to internal flaws, such as cracks and cavities [12,13]. Especially in applications, PSC devices are usually subjected to a multi-physics field such as mechanical, electrical and thermal loads. Such loadings concentrated at flaws may produce a coupling stress, which causes mechanical or electrical degradation and even failure of devices. In view of the reliability of PSC-based devices in multi-field working environments, their fracture properties have attracted considerable research attention. For example, Yang [14] considered a semi-infinite crack and found out that there are certain differences in fracture behavior of PSC from insulating piezoelectric materials, and furthermore, obtained an analytical solution for both stress and electric fields near a crack. Hu [15] analyzed the singularities of physics fields at a type-III crack tip in PSC, and presented that the fracture behavior is closely related to the semiconductor properties. Sladek et al. [16] and Lu et al. [17] investigated the dynamic anti-plane crack in functional graded PSC, derived local integral equations that involve one order lower derivatives than the original partial differential equations, and finally, built up a system of ordinary differential equations for the involved nodal unknown quantities. In addition, they discovered that the stress and electric displacement fields in PSC exhibit the same singularities, and recently, they studied the three-dimensional functionally graded PSC beam using meshless method and showed that the material parameter gradation and initial electron density have a large influence on intensity factors of cracks in PSC materials. Based on the extended displacement discontinuity boundary integral equation, Zhao et al. [18] numerically simulated and verified the stress singularities near the edge of a planar crack. According to the finite element and boundary element methods, Fan et al. [19] and Zhang et al. [20] proposed a piezoelectric-conductor iterative approach for structural analysis of PSC with combined mechanical and electrical loading. Zhao et al. [21] analyzed a penny-shaped crack in the isotropic plane of PSC and, based on Almansi’s theory, derived its general solutions.

To develop the fracture criterion of PSCs, many theoretical efforts have been done. Usually, the fracture criterion of insulating dielectric ceramics [22,23,24] is directly applied to analyze the multi-field fracture of PSCs, in which the effect of electric current field was ignored. The fracture process is thought to be controlled only by mechanical and electric field intensity factors. However, it is difficult to judge such a method because there is no available experimental evidence. Therefore, an urgent need is to experimentally investigate the effect of electric current on fracture of PSCs under a combined mechanical and electrical load and determine its fracture criterion, which can provide a theoretical basis for improving the reliability of PSC devices.

The paper is structured as follows. The experimental procedure is described in Section 2, and in Section 3, finite element analysis is performed to obtain the expression of intensity factors. Then, based on the experimental and numerical results, the fracture behavior and fracture criterion are discussed in Section 4. Finally, the main conclusions are summarized in Section 5.

## 2. Experiment

### 2.1. Material and Specimens

**Figure 1:**
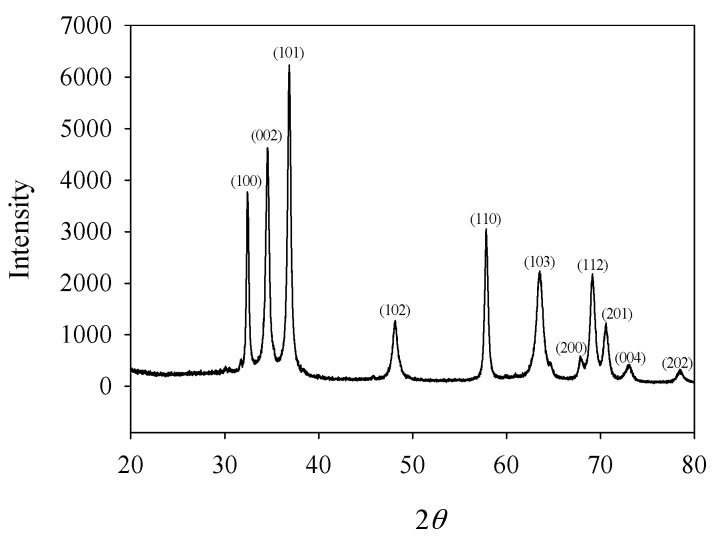
X-ray diffraction pattern of a polarized GaN sample.

The material that is used in this work is GaN, which is a new kind of electronic ceramics materials with piezoelectric and semiconductor properties. Due to the stable conductivity and excellent functional properties, GaN is a perfect candidate material of electromechanical, high-power and high-frequency devices for operation in extreme environments [25,26,27,28]. Here, GaN ceramics were manufactured from pure GaN powder by vacuum hot pressing at 450 °C (with a density of 5.9 relative to water), which exhibits properties similar to that of *N*-type semiconductors with a Curie temperature of 265 °C. It is seen from Figure 1 that the X-ray diffraction pattern matches well with the standard spectrum of JCPDS file (i.e., No. 76-0703) of GaN.

**Figure 2:**
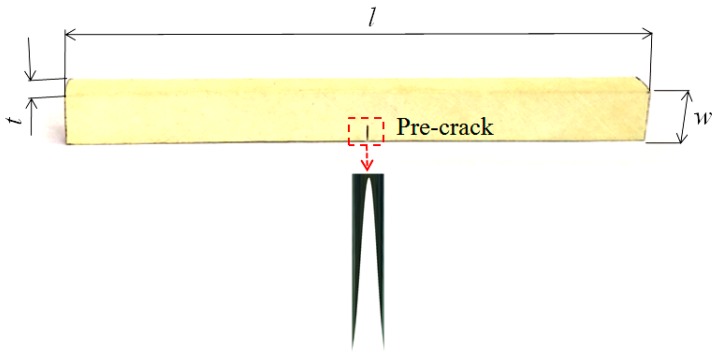
A standard three-point bending specimen for fracture test.

In accordance with the three-point bending fracture test standard of fine ceramics [29], single-edge notched beam (SENB) specimens were manufactured. The dimensions of these samples are illustrated in Figure 2, with the sample length *l* = 40.0 mm, thickness *t* = 3.0 mm, and width *w* = 4.0 mm. Specimens were polarized along its thickness direction using a polarization field of 32 kV cm^−1^ (the coercive field *E*_c_ = 8.19 kV cm^−1^). Here, it is worth noting that polarization along the length direction of a sample needs a higher voltage, which will be done in our next work. The poling temperature and time were 120 °C and 30 min. During the poling process, two poly tetra fluoro ethylene sheets (PTFE) were bond on both surfaces along the thickness direction of a sample with a thickness of PTFE *t*_s_ = 0.5 mm. Then, silver paste was plated on the up and bottom surfaces of PTFE sheets. An adjustable DC high-voltage (provided by a power supply of 100 kV) was applied to silver wires that were welded on electrodes. All of the polarization jigs were placed in a plexiglass container that was filled with silicone oil (with a relative dielectric constant of 2.73) to prevent high-voltage discharging. Under an applied electric field (32 kV cm^−1^), carriers in a sample would be redistributed (electrons accumulate the positive pole and holes towards to the negative pole), and a new internal electric field would be generated, which can cause domain switch and finally make the sample reach the saturation polarization strength. After polarizing, the samples were cleaned by an ultrasonic cleaner and stored in a drum wind drying oven at 70 °C for 10 min.

To minimize the notch passivation effect [30], a pre-notch was cut using an automatically precise incises machine with a very thin circle saw blade that was made of diamond and bronze (0.07 mm). The crack length (*a*) ranges from 1.43 mm to 2 mm, and at the bottom of a pre-crack, there is an arc with a diameter between 20 μm and 60 μm (see Figure 2).

### 2.2. Experimental Configuration and Fracture Tests

**Figure 3:**
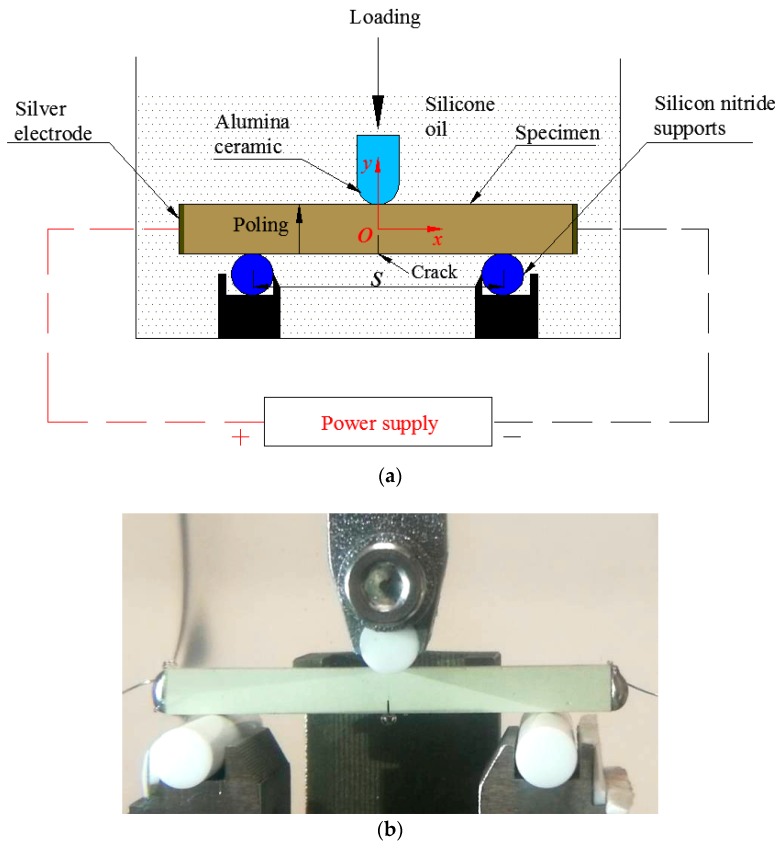
(**a**) Schematic representation of an experimental configuration and (**b**) an actual coupling experimental loading structure.

As shown in Figure 3, a multi-field three-point bending fracture system was constructed with the span *s* = 30.0 mm. While considering insulation requirements during mechanical loading, the loading head and supports were made of alumina ceramic and silicon nitride ceramic, respectively. In addition, all the jigs were fixed in a plexiglass container that was filled using silicone oil to prevent electrical sparking. Both ends of a sample length were plated with silver, a viscous emulsion that solidified quickly at 200 °C, to act as electrodes. Then, silver wires were welded at the left and right sides of a sample using an electric iron to connect the power supply.

During three-point bending fracture tests, the mechanical load *P* was increased monotonically at a rate of 0.05 mm min^−1^. The electric current was applied by a linear power that can be adjusted with a high resolution of 10^−4^ A. The critical mechanical and electrical loads at fracture were recorded to calculate the physical field and critical intensity factors, so as to investigate the relationship between the applied current and the fracture behaviors of PSC. Fracture morphologies were investigated via an ultra deep field microscope (KEYENCE, VHX-700FC, Osaka, Japan).

## 3. Numerical Analysis

Generally, it is difficult to study the fracture behaviors of materials that were subjected to coupled loading. To further clarify the fracture characteristics of PSCs under a combined electrical and mechanical load, we have to resort to numerical analysis, and thus, a numerical model together with finite element analysis was developed.

### 3.1. Basic Equations

An *oxy* Cartesian coordinate system was set up, with the origin point *o* at a sample center and the *x* and *y* being symmetrical axes, as illustrated in Figure 3. For a plane problem of PSCs with an *N*-type semiconductor property, the linearized equilibrium equations were given by Hu et al. [15], that is

(1a) $$\begin{array}{l} \frac{\partial \sigma_{xx}}{\partial x}+\frac{\partial \sigma_{xy}}{\partial y}=0,\\ \frac{\partial \sigma_{xy}}{\partial x}+\frac{\partial \sigma_{yy}}{\partial y}=0, \end{array}$$

(1b) $$\frac{\partial D_{x}}{\partial x}+\frac{\partial D_{y}}{\partial y}=-q\Delta n\;$$

(1c) $$\frac{\partial J_{x}}{\partial x}+\frac{\partial J_{y}}{\partial y}=0,\;$$

where *σ_ij_* are the stress components (*i*, *j* = *x*, *y*), *D_i_* and *J_i_* are the components of electric displacement vector and electrical current, respectively, *q* is the elementary charge, and Δ*n* is the variation of carrier density.

The constitutive relation of two-dimensional PSC (*N*-type) with its polarization direction along the *y*-direction is as follows [31,32]:

(2a) $$\begin{array}{l} \sigma_{xx}=c_{11}\frac{\partial u}{\partial x}+c_{13}\frac{\partial v}{\partial y}+e_{31}\frac{\partial \phi }{\partial y},\\ \sigma_{yy}=c_{13}\frac{\partial u}{\partial x}+c_{33}\frac{\partial v}{\partial y}+e_{33}\frac{\partial \phi }{\partial y},\\ \sigma_{xy}=c_{44}(\frac{\partial u}{\partial y}+\frac{\partial v}{\partial x})+e_{15}\frac{\partial \phi }{\partial x}, \end{array}$$

(2b) $$\begin{array}{l} D_{x}=e_{15}(\frac{\partial u}{\partial y}+\frac{\partial v}{\partial x})-\kappa_{11}\frac{\partial \phi }{\partial x},\\ D_{y}=e_{31}\frac{\partial u}{\partial x}+e_{33}\frac{\partial v}{\partial y}-\kappa_{33}\frac{\partial \phi }{\partial y}, \end{array}$$

(2c) $$\begin{array}{l} J_{x}=-qn_{0}\mu_{11}\frac{\partial \phi }{\partial x}+qd_{11}\frac{\partial \Delta n}{\partial x},\\ J_{y}=-qn_{0}\mu_{33}\frac{\partial \phi }{\partial y}+qd_{33}\frac{\partial \Delta n}{\partial y}, \end{array}$$

where *u* is the elastic displacement in the *x*-direction and *v* is the elastic displacement in the *y*-direction, *φ* is the electric potential. *c_ij_*, *e_ij_*, and *κ_ij_* are the elastic, piezoelectric, and dielectric constants. *n*_0_ is the initial carrier density, and *μ_ij_* and *d_ij_* are the electron mobility and diffusion, respectively.

By substituting Equation (2) into Equation (1), the governing equations of PSC can be obtained as

(3a) $$\begin{array}{l} (c_{11}\frac{\partial^{2}}{\partial x^{2}}+c_{44}\frac{\partial^{2}}{\partial y^{2}})u+(c_{13}+c_{44})\frac{\partial^{2}v}{\partial x\partial y}+(e_{15}+e_{31})\frac{\partial^{2}\phi }{\partial x\partial y}=0,\\ (c_{13}+c_{44})\frac{\partial^{2}u}{\partial x\partial y}+(c_{44}\frac{\partial^{2}}{\partial x^{2}}+c_{33}\frac{\partial^{2}}{\partial y^{2}})v+(e_{15}\frac{\partial^{2}}{\partial x^{2}}+e_{33}\frac{\partial^{2}}{\partial y^{2}})\phi =0, \end{array}$$

(3b) $$(e_{15}+e_{31})\;\frac{\partial^{2}u}{\partial x\partial y}+(e_{15}\frac{\partial^{2}}{\partial x^{2}}+e_{33}\frac{\partial^{2}}{\partial y^{2}})v-(\kappa_{11}\frac{\partial^{2}}{\partial x^{2}}+\kappa_{33}\frac{\partial^{2}}{\partial y^{2}})\phi =-q\Delta n,\;$$

(3c) $$q\;(d_{11}\frac{\partial^{2}}{\partial x^{2}}+d_{33}\frac{\partial^{2}}{\partial y^{2}})\Delta n=qn_{0}(\mu_{11}\frac{\partial^{2}}{\partial x^{2}}+\mu_{33}\frac{\partial^{2}}{\partial y^{2}})\phi .\;$$

### 3.2. Boundary Conditions

Under the experimental conditions (see Figure 3), the boundary conditions at the right and left sides of a sample can be written as [33,34]

(4) $$\begin{array}{l} \sigma_{xx}=0,\;\sigma_{xy}=0,\;\phi =V_{a}+V_{bi},\;J_{x}=qv_{\mathrm{rec}}^{}(n-n_{m}),\;x=-l/2,\\ \sigma_{xx}=0,\;\sigma_{xy}=0,\;\phi =V_{bi},\;J_{x}=-qv_{\mathrm{rec}}(n-n_{m}),\;x=l/2, \end{array}$$

where *V*_a_ is the voltage applied at the left end, *ν*_rec_ is the thermal recombination velocity, and *n*_m_ is the critical electron density that can be expressed as

(5) $$n_{m}=N_{c}e^{-\Phi_{B}/k_{B}T},\;$$

(6) $$N_{c}=2{\left(\frac{2\pi \;m_{e}k_{B}\;T}{h^{2}}\right)}^{3/2},\;$$

where Φ_B_ is the surface barrier of GaN, *T* is absolute temperature, *k*_B_ is the Boltzmann constant, *h* is the Planck constant, and *m*_e_ = 1.82 × 10^−31^ kg and *N*_c_ = 2.23 × 10^24^ m^−3^ denotes the effective mass of a conduction band electron and the effective state density of conduction bands, respectively. *V*_bi_ is the built-in voltage [34,35] that is given by

(7) $$V_{\mathrm{bi}}=\Phi_{B}-V_{\mathrm{th}}\ln(N_{c}/n_{0}),\;$$

(8) $$V_{\mathrm{th}}=k_{B}T/q,\;$$

The barrier height was formed by Schottky contact [34,35] and it is determined by

(9) $$q\Phi_{B}=q(\Phi_{M}-\chi ),\;$$

which represents the difference between the working function of silver, *q*Φ_M_ (4.26 eV) [36], and the electron affinity of GaN, *qχ* (4.10 eV) [37]. Therefore, the potential barrier can be calculated (0.16 V).

At the three contact points, the boundary conditions are given by

(10) $$\begin{array}{l} F_{y}=-P,\;u=0,\;x=0,y=w/2,\\ v=0,\;x=\pm \;s/2,\;y=-w/2. \end{array}$$

On the crack faces, the insulating boundary conditions are adopted as [38]

(11) $$\sigma_{ij}l_{j}=0,\;D_{j}l_{j}=0,\;J_{j}l_{j}=0,\;$$

where *l_j_* is the outer normal vector.

### 3.3. Intensity Factor

By means of COMSOL Multiphysics software, finite element analysis was performed to calculate the stress intensity factor, *K_σ_*, the electric displacement intensity factor, *K_D_*, and the electric current intensity factor, and *K_J_*, for the given sample geometry and loading conditions (see Figure 3). The material parameters of polarized GaN PSCs were listed in Table 1 [39].

**Figure 4:**
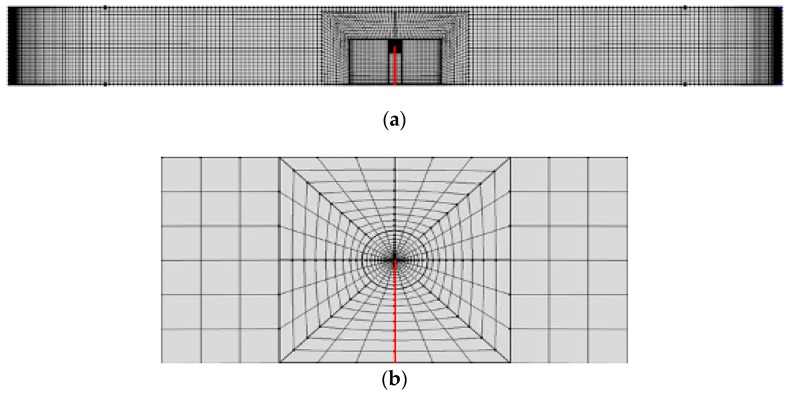
(**a**) The finite element mesh for a PSCs specimen and (**b**) its locally refined meshes at the crack tip.

The finite element model of a specimen is shown in Figure 4. In order to obtain numerical results with a high accuracy, very fine meshes were organized near the crack tip. In addition, meshes that were used in the Schottky contact boundary layer were similarly refined. According to the literature [40], the element size should be less than the Debye length, *L*_D_, that is,

(12) $$L_{D}=\sqrt{\frac{\kappa_{11}k_{B}T}{q^{2}n_{0}}}.\;$$

We have *L*_D_ = 10.5 nm by substituting the relevant constants into formula above. In finite element analysis, the minimum mesh size of 8 nm was applied near the Schottky contact surface.

According to Zhao et al. [18], all the stress, electric displacement, and electric current densities near the crack tip have a classical singular index of 1/2. Therefore, we can define the three intensity factors of PSCs (*K_σ_*, *K_D_*, and *K_J_*), as follows:

(13a) $$K_{\sigma }=\underset{r\to 0}{\lim}\sigma_{xx}(0,r+a-w/2)\sqrt{2\pi r},$$

(13b) $$K_{D}=\;\underset{r\to 0}{\lim}D_{x}(0,r+a-w/2)\sqrt{2\pi r},$$

(13c) $$K_{J}=\;\underset{r\to 0}{\lim}\;J_{x}(0,r+a-w/2)\sqrt{2\pi r},$$

where *r* is the distance from the crack tip in the *y*-direction.

For simplification and comparison, these intensity factors are normalized as

(14a) $$F_{\sigma }=K_{\sigma }/K_{\sigma }^{0},\;$$

(14b) $$F_{D}=K_{D}/K_{D}^{0},\;$$

(14c) $$F_{J}=K_{J}/K_{J}^{0},\;$$

where *K_σ_*^0^, *K_D_*^0^, and *K_J_*^0^ are the three intensity factors in the case of *N*_D_ = 1.29 × 10^23^ m^−3^, *P* = 4 N, and *E*_a_ = 10^6^ V m^−1^.

**Figure 5:**
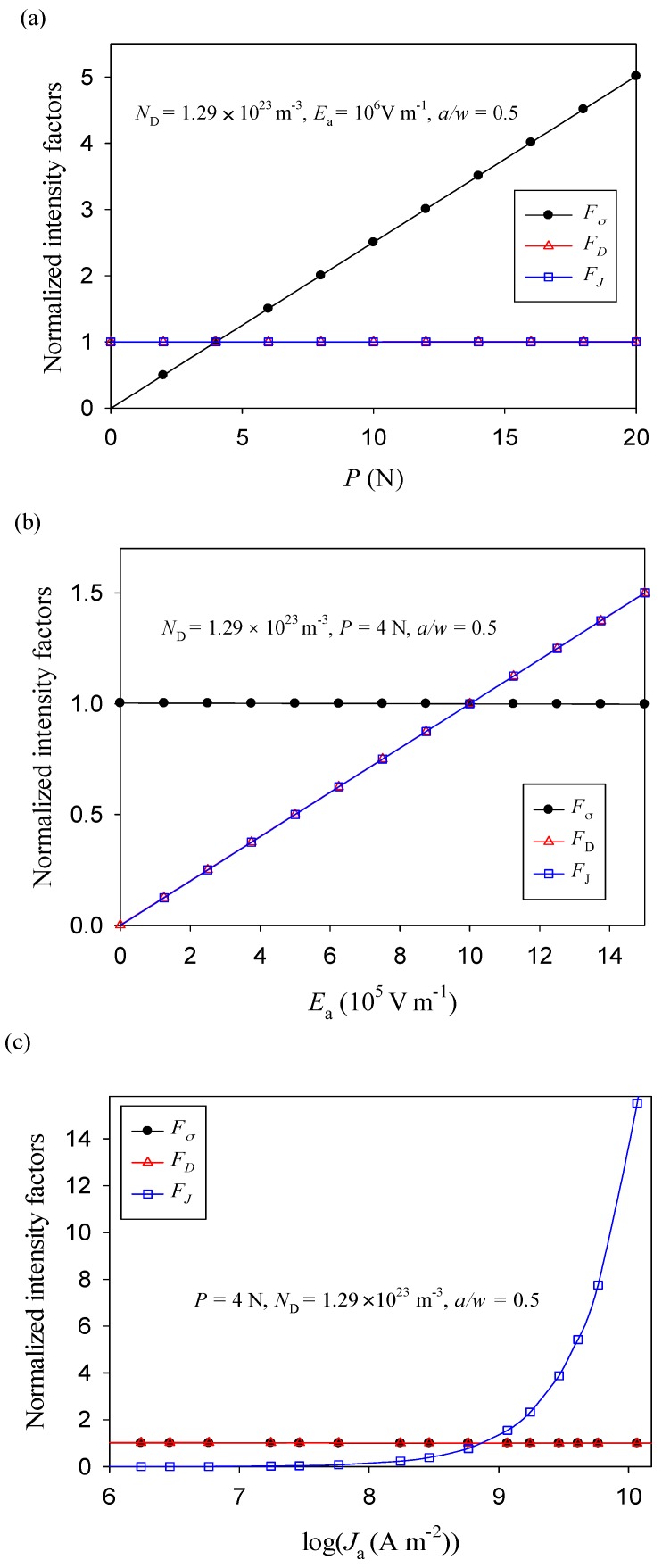
Normalized intensity factors versus loads under (**a**) the mechanical loading, (**b**) the electric field, and (**c**) the electric current density.

As shown in Figure 5, the stress intensity factor is solely related to the applied mechanical load, the electric displacement intensity factor is only connected with the applied electric field strength, and the electric current intensity factor is just dependent on the applied electric current and the accompanying electric field. This is because the polarization direction of a specimen is oriented along the *y*-axis, and thus it is perpendicular to the direction of an applied electric field (in the *x*-direction), which is uncoupled with the stress component *σ_xx_* (see Equation (2a)). Therefore, the electric load has no effect on the stress intensity factor. Similarly, due to the polarization direction along the *y*-axis, the piezoelectric charge moves in the *y-*axis direction under the applied mechanical load (piezoelectric-semiconductive effect). The electric current density in the *x-*direction (*J_x_*) and the electric displacement in the *x*-direction (*D_x_*) are not affected by the mechanical load (see Figure 5).

**Figure 6:**
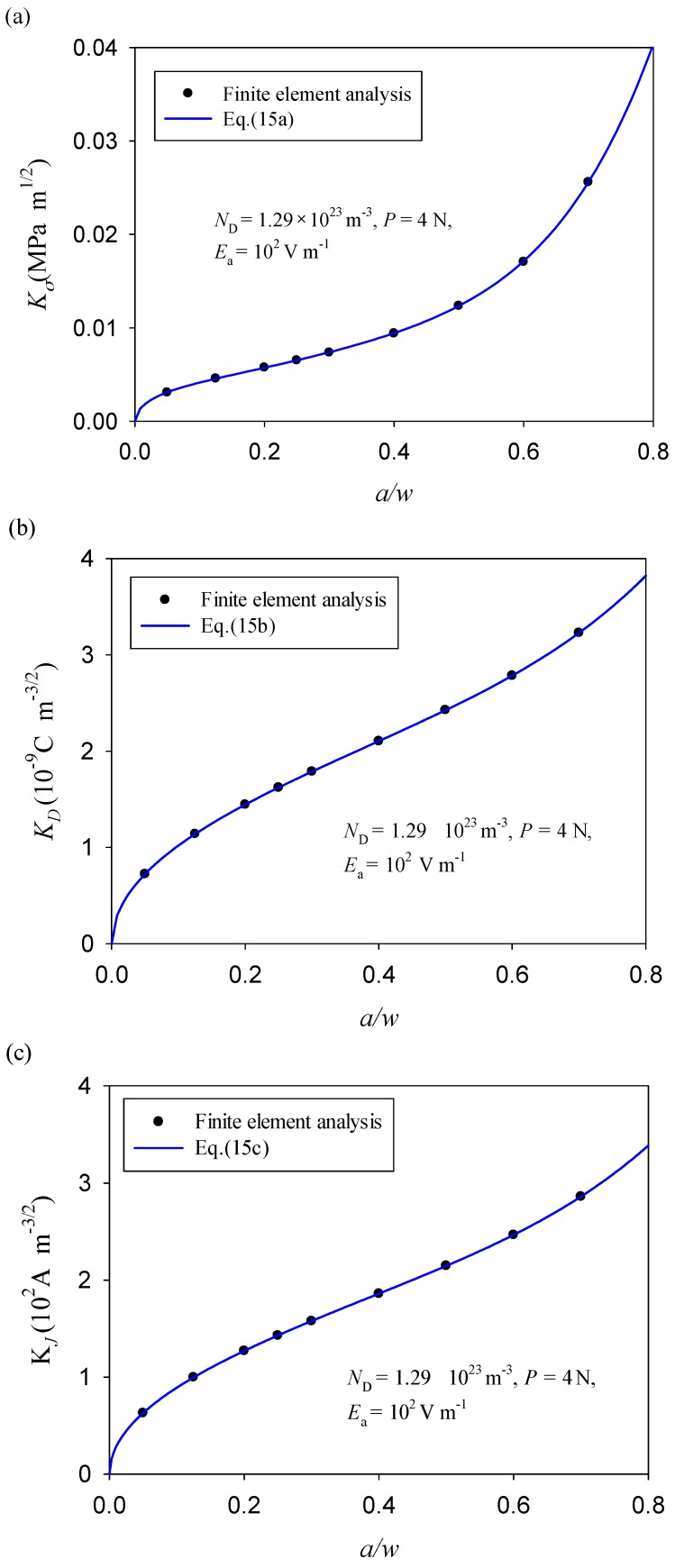
The fitted empirical formulae of the intensity factors of GaN PSCs, (**a**) stress intensity factor, (**b**) electric displacement intensity factor and (**c**) electric current intensity factor.

With reference to the standard form (ISO15732:2003) [29], the formulas of intensity factors were fitted by using the numerical results for specimens with a single-edge crack, ranging from 1.43 mm to 2 mm. As shown in Figure 6, no matter how the crack length changes (*a/w*), the the stress intensity factor, *K_σ_*, the electric displacement intensity factor, *K_D_*, and the electric current intensity factor, and *K_J_*, conform to a unique fitting function (see Figure 6a–c), that is

(15a) $$K_{\sigma }=f_{1}(\frac{a}{w})\frac{Ps}{tw^{3/2}},\;$$

(15b) $$K_{D}=f_{2}(\frac{a}{w})\kappa_{11}E_{a}w^{1/2},\;$$

(15c) $$K_{J}=f_{3}(\frac{a}{w})J_{a}w^{1/2},\;$$

with

(16) $$f_{i}\left(\frac{a}{w}\right)=\sum_{j=1}^{5}e_{j}{\left(\frac{a}{w}\right)}^{\frac{2j-1}{2}}\;$$

where *P* is the mechanical load. *E*_a_ and *J*_a_ represent the applied electric field and electric current density, respectively. *f_i_* (*a/w*) are the shape factors of a three-point bending specimen, and *i* = 1, 2, and 3. *e_j_* are the fitted coefficients, which are corresponding to the stress, electric displacement, and electric current intensity factors, respectively, as summarized in Table 2.

## 4. Results and Discussion

**Figure 7:**
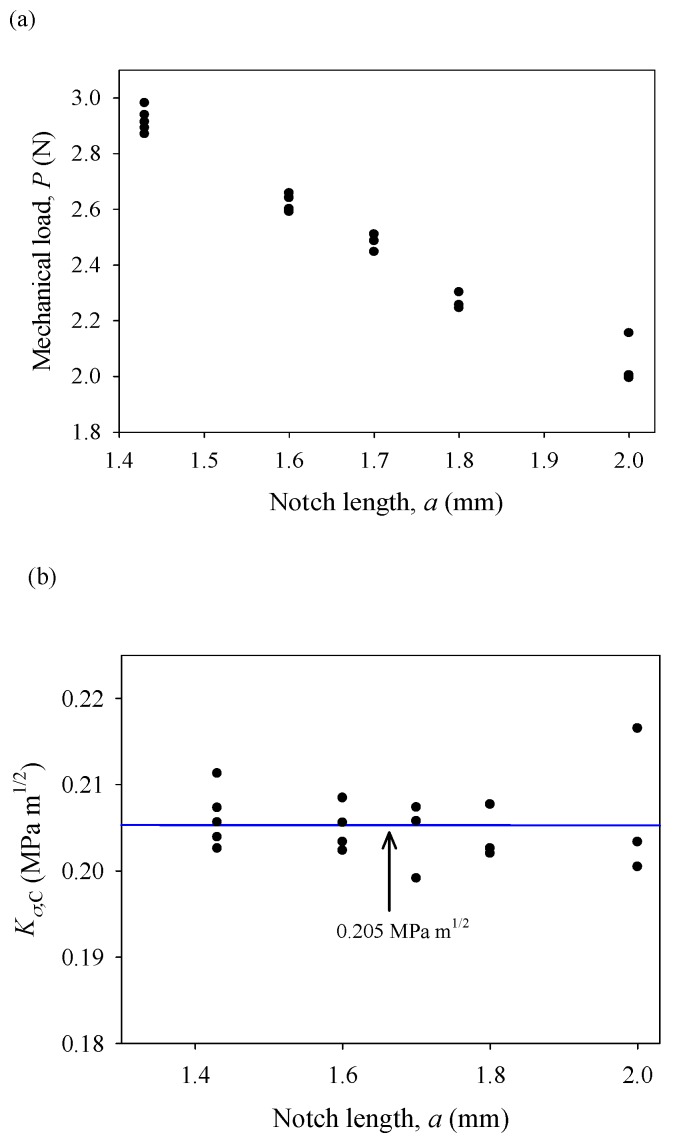
(**a**) Critical mechanical load versus the length of a pre-crack under a pure mechanical load and (**b**) the critical stress intensity factors of GaN PSCs.

As shown in Figure 7a, the critical load at fracture decreases linearly as the crack length increases under a purely mechanical load. Based on critical loads and Equation (15a), the critical stress intensity factors were calculated and are shown in Figure 7b. It is seen that the critical stress intensity factor (fracture toughness) is *K_σ_*_,*C*_ = 0.205 ± 0.011 MPa m^1/2^, which is obviously lower than fracture toughness of insulating piezoelectric ceramics (around 1 MPa m^1/2^) [41,42,43]. As is well known, fracture toughness is a material property that can serve as a fracture criterion. Thus, we need to further investigate fracture under combined mechanical and electrical loading and verify the influence of electric current on the fracture behavior of conductive PSCs.

**Figure 8:**
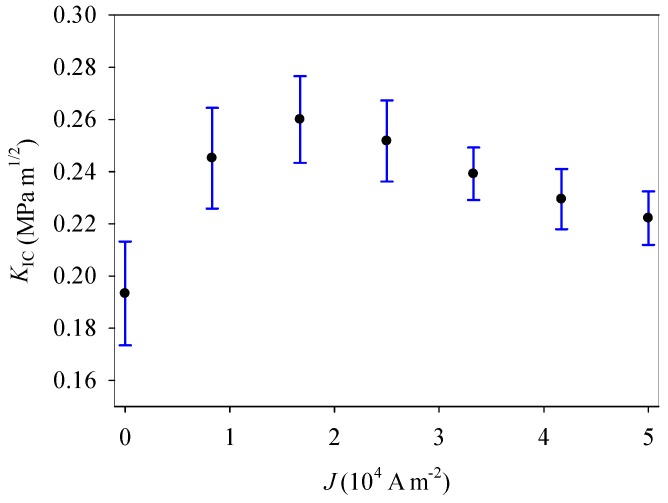
The relationship of *K*_IC_ with the applied electric current density.

**Figure 9:**
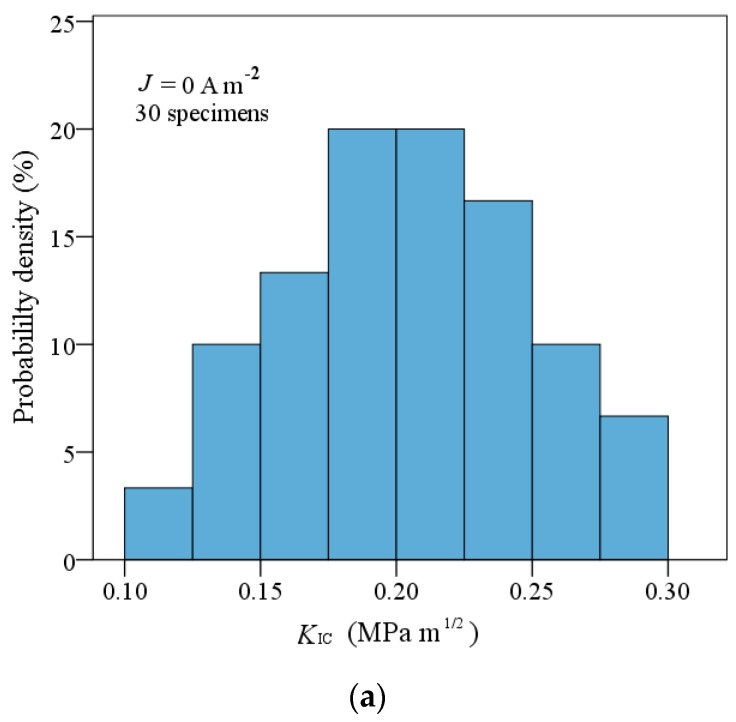

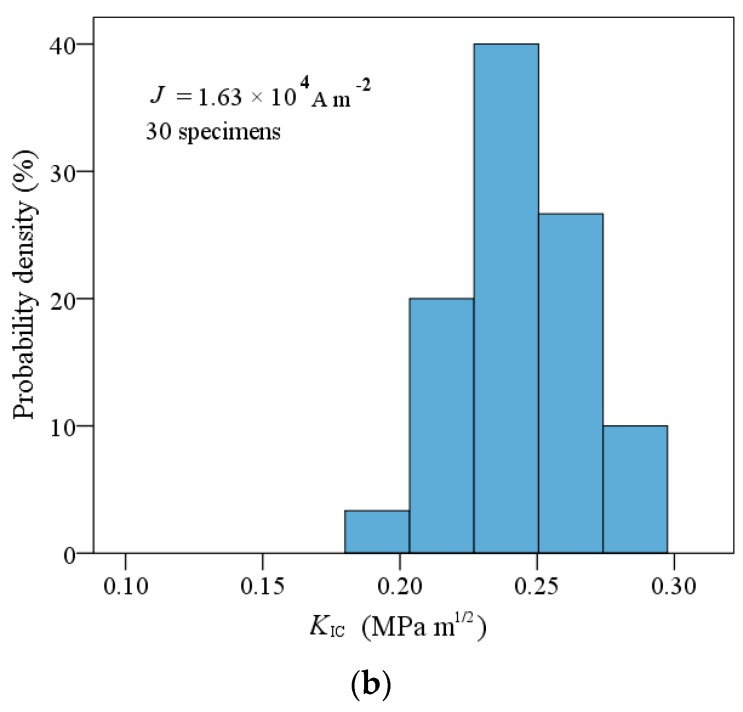
The probability density of *K*_IC_ under (**a**) the pure mechanical loading and (**b**) the combined electrical and mechanical loading (1.63 × 10^4^ A m^−2^).

It is seen from Figure 8 that, as the applied electric current density increases from 0 to 1.63 × 10^4^ A m^−2^, the mean value of the critical stress intensity factor increases from 0.19 to 0.26 MPa m^1/2^, and thus fracture toughness increases by 36.8%. However, as the applied electric current further increases, fracture toughness decreases. Under an applied electric current, the corresponding fracture toughness distributions can be completely changed (see Figure 9). That is, the fracture behavior of PSCs is significantly influenced by an electric current. Therefore, under combined mechanical and electric loading, electric current should be introduced in the fracture criteria for PSCs with a single-edge crack.

**Figure 10:**
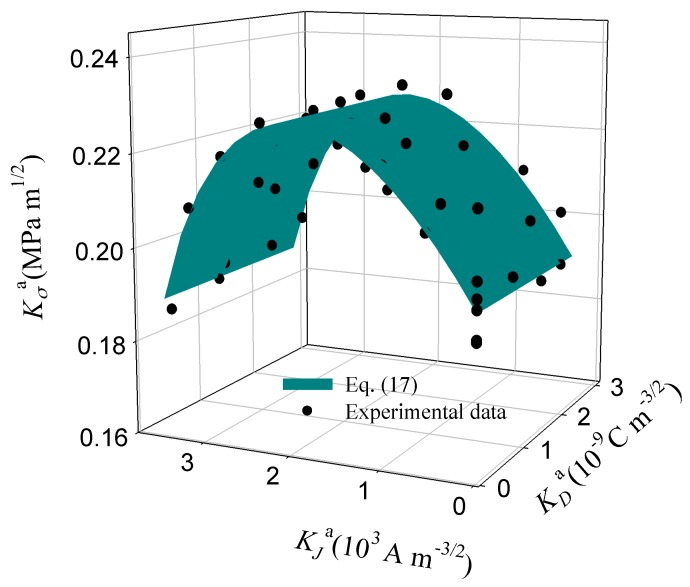
Experimental and fitting results for failure of PSCs specimens with a single-edge crack under combined mechanical and negative electrical loading.

Here, it is worth noting that the fracture criterion of an insulating piezoelectric ceramic can be expressed as an ellipse of two independent variables, i.e., the stress and electric field intensity factors [44,45]. According the experimental results, fracture of conductive PSCs is related to the stress, electric displacement, and electric current. Therefore, as shown in Figure 10, the fracture criterion can be represented by the following ellipsoidal expression, that is

(17) $$\begin{array}{l} {\left(\frac{K_{\sigma }^{a}}{K_{\sigma ,C}}\right)}^{2}+{\left(\frac{K_{D}^{a}}{K_{D,C}}\right)}^{2}+{\left(\frac{K_{J}^{a}}{K_{J,C}}\right)}^{2}+d\left(\frac{K_{\sigma }^{a}}{K_{\sigma ,C}}\right)\left(\frac{K_{D}^{a}}{K_{D,C}}\right)\\ +f\left(\frac{K_{D}^{a}}{K_{D,C}}\right)\left(\frac{K_{J}^{a}}{K_{J,C}}\right)+g\left(\frac{K_{J}^{a}}{K_{J,C}}\right)\left(\frac{K_{\sigma }^{a}}{K_{\sigma ,C}}\right)=1. \end{array}$$

where the fitted critical stress intensity factor *K_σ,C_* = 0.194 MPa m^1/2^, the critical electric displacement intensity factor *K_D_*_,*C*_ = 3.409 × 10^13^ C m^−3/2^, the critical current density intensity factor *K_J_*_,*C*_ = 3.236 × 10^3^ A m^2^, with the normalized fitting coefficients *d* = 51.65, *f* = 51.64, and *g* = −1.05. The mechanical and electrical fracture toughness of PSC GaN was obtained. Due to the theoretical simplification and scattering of the experimental data, there are differences between the theoretical and experimental results (see Figure 10). Nevertheless, it is indicated that Equation (17) is able to serve as a failure criterion for PSC specimens with a single-edge crack under a combined electric current, electric field, and mechanical load. The fracture behavior of GaN PSCs can be predicted when the geometry conditions and information of loads are available.

**Figure 11:**
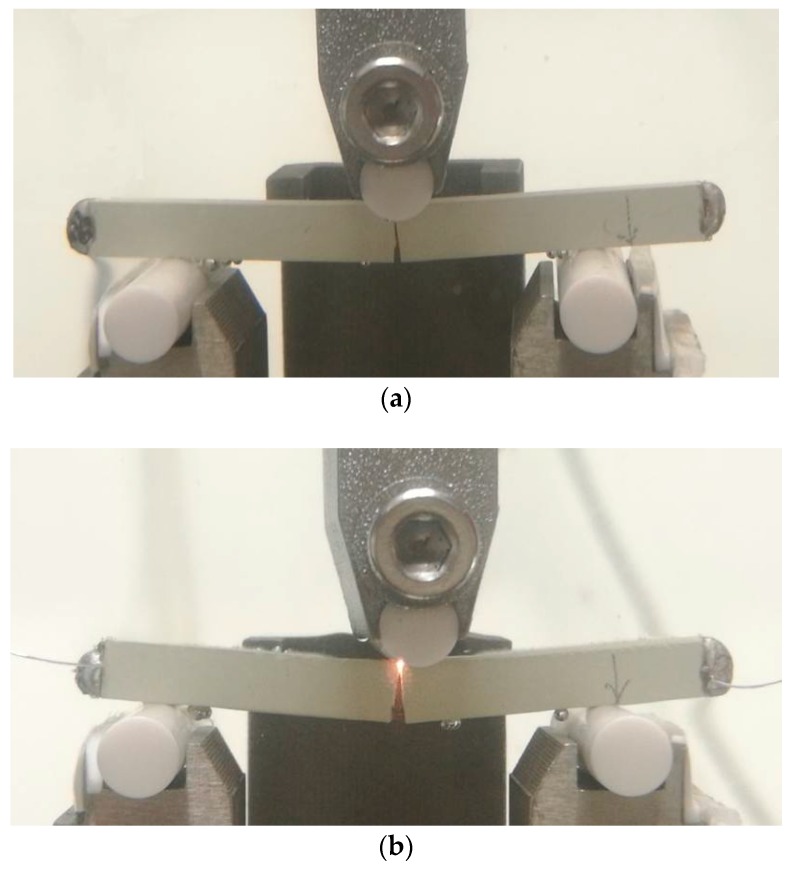
Specimens in fracture testing (**a**) without an applied electric current and (**b**) under the combined electrical and mechanical loading (1.63 × 10^4^ A m^−2^).

**Figure 12:**
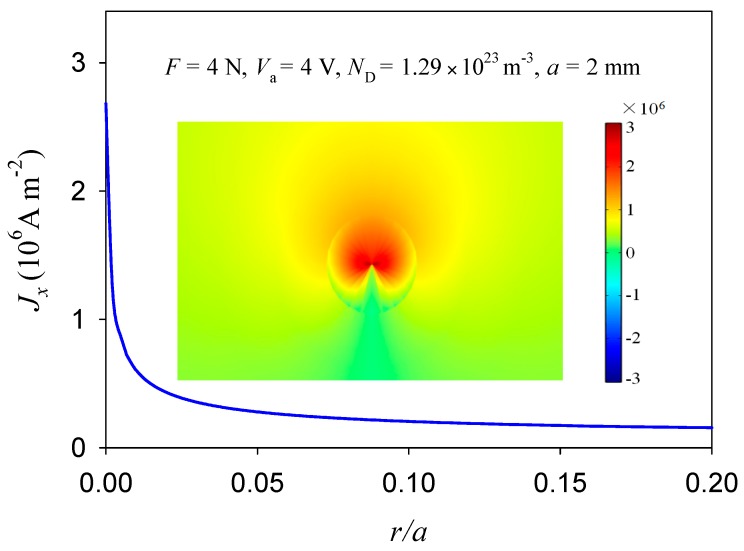
The electric current density distribution in the extension of a crack tip (see Figure 2) and the corresponding nephogram near the crack tip.

**Figure 13:**
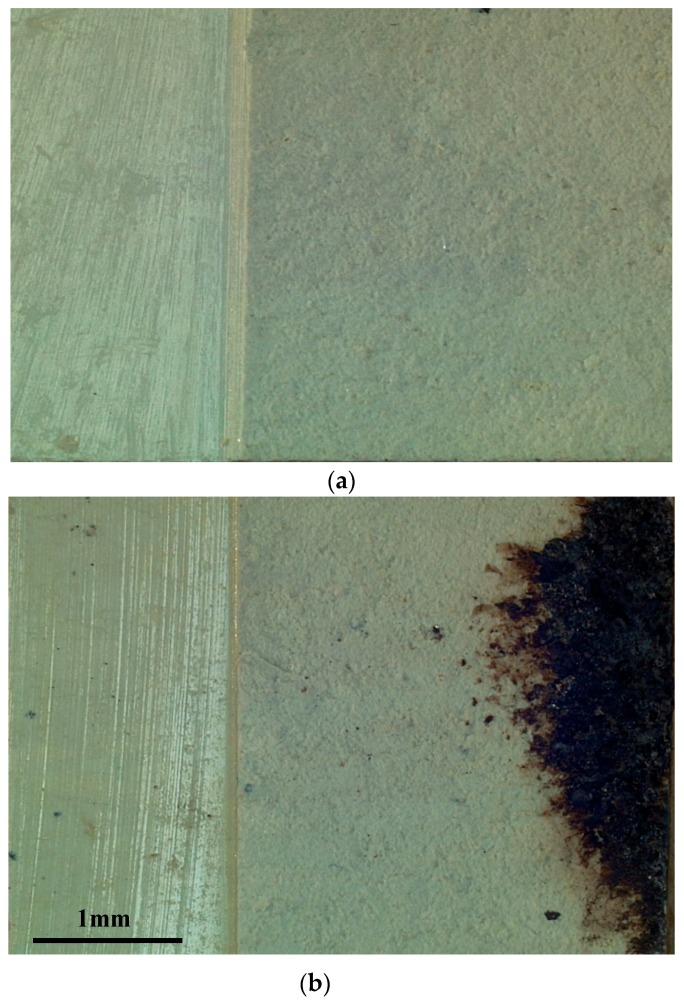
Fracture morphologies under (**a**) the pure mechanical loading and (**b**) the combined electrical and mechanical loading.

When compared to purely mechanical loading, there is an obvious discharge near the crack tip under a combined electrical and mechanical load (see Figure 11). This is due to the electric current concentrate at a crack tip that causes local discharge. Numerical results show that the electric current density at the crack tip reached a value of 10^6^ A m^−2^ (see Figure 12). In case of pure mechanical load, fracture surface was flat, as shown in Figure 13a. Under combined electrical and mechanical loading, however, fracture surface was rough and its most area was melted and re-solidified. The difference in the shape of fracture surfaces is due to defects that are sensitive to the electric current. The concentrated electric current can cause discharge at the crack tip and burn the extension surface, as seen from the dark area in Figure 13b.

## 5. Conclusions

The fracture of PSCs under combined mechanical, voltage, and current loading was investigated by using a three-point bending experiment method. The experimental results show that the electric current density is a key factor to the fracture behavior of PSCs, and thus, an electric current density factor should be involved in the fracture criterion. The expressions of stress, electric displacement, and electric current density intensity factors of PSCs were obtained via finite element analysis. On this basis, a fracture criterion under combined electrical and mechanical loading was established by fitting the experimental data.

It is shown that, given that the relevant information on sample geometries and material properties such as mechanical and electrical fracture toughness, it is possible to predict the critical loads and vice versa. It is expected that the fracture criterion and the discovery of new material properties can be helpful in greatly improving the reliability and service life of PSCs, and to reduce use-cost, especially for the derating design of GaN electro-mechanical coupling devices. Finally, it is also worth noting that, to clarify electric current-based mechanisms and consider different polarization and electric field directions, further experimental and theoretical works are still needed.

## Figures and Tables

**Table 1:** Material parameters of polarized GaN piezoelectric semiconductor ceramics (PSCs).

Elastic Stiffness (10^9^ Nm^−2^)	Piezoelectric Constant (C m^−2^)	Relative Dielectric Constant (*k_ij_*/*k*_0_)	Migration Rate (cm^2^ V^−1^ s^−1^)	Diffusion Coefficient (cm^2^ s^−1^)
*C*_11_= 298.4	*e*_31_ = −0.52	*ε*_11_ = 9.5	*μ*_11_ = 653	*d*_11_ = 16.99
*C*_12_ = 121.0	*e*_15_ = −0.31	*ε*_33_ = 10.3	*μ*_33_ = 982	*d*_33_ = 25.53
*C*_13_ = 142.5	*e*_33_ = 0.61			
*C*_33_ = 289.2				
*C*_44_ = 23.1				

**Table 2:** Fitting coefficients (*e_j_* in Equation (16)) under the different types of intensity factors.

Intensity Factors	Fitting Coefficients, *e_j_* (*j* = 1, 2, 3, 4, 5)				
Stress intensity factor	3.055	−7.141	33.479	−61.360	52.909
Electric displacement intensity factor	1.921	−0.426	2.157	−3.438	3.206
Electric current intensity factor	1.800	−0.256	2.350	−4.101	4.270

## References

[1] Yang G., Du J.K., Wang J., Yang J.S. (2018). Electromechanical fields in a nonuniform piezoelectric semiconductor rod. J. Mech. Mater. Struct..

[2] Qin G.S., Zhang X., Ma S.J., Zhang Q.Y., Fan C.Y., Zhao M.H. (2018). An accurate computational method for analysis of electromechanical properties of structures with metal-GaN piezoelectric semiconductor contact. Comput. Mater. Sci..

[3] Fan S.Q., Liang Y.X., Xie J.M., Hu Y.T. (2017). Exact solutions to the electromechanical quantities inside a statically-bent circular ZnO nanowire by taking into account both the piezoelectric property and the semiconducting performance: Part I—Linearized analysis. Nano Energy.

[4] Hutson A.R. (1960). Piezoelectricity and conductivity in ZnO and CdS. Phys. Rev. Lett..

[5] Heyman J.S. (1978). Phase insensitive acoustoelectric transducer. J. Acoust. Soc. Am..

[6] Busse L.J., Miller J.G. (1981). Response characteristics of a finite aperture, phase insensitive ultrasonic receiver based upon the acoustoelectric effect. J. Acoust. Soc. Am..

[7] Dietz D.R., Busse L.J., Fife M.J. (1988). Acoustoelectric detection of ultrasound power with composite piezoelectric and semiconductor devices. IEEE Trans. Ultrason. Ferroelectr..

[8] Hickernell F.S. (2005). The piezoelectric semiconductor and acoustoelectronic device development in the sixties. IEEE Trans. Ultrason. Ferroelectr..

[9] Jones K.A., Chow T.P., Wraback M. (2015). AlGaN devices and growth of device structures. J. Mater. Sci..

[10] Wen X.N., Wu W.Z., Pan C.F., Hu Y.F., Yang Q., Wang Z.L. (2015). Development and progress in piezotronics. Nano Energy.

[11] Trotta R., Wildmann J.S., Zallo E., Schmidt O.G., Rastelli A. (2014). Highly entangled photons from hybrid piezoelectric-semiconductor quantum dot devices. Nano Lett..

[12] Alamo J.A.D., Joh J. (2009). GaN HEMT reliability. Microelectron. Reliab..

[13] Farrer J.K., Carter C.B. (2006). Defect structure in GaN pyramids. J. Mater. Sci..

[14] Yang J.S. (2005). An anti-plane crack in a piezoelectric semiconductor. Int. J. Fracture.

[15] Hu Y.T., Zeng Y., Yang J.S. (2007). A mode III crack in a piezoelectric semiconductor of crystals with 6 mm symmetry. Int. J. Solids Struct..

[16] Sladek J., Sladek V., Pan E. (2014). Dynamic anti-plane crack analysis in functional graded piezoelectric semiconductor crystals. Cmes-Comp. Model. Eng..

[17] Lu H.H., Young D.L., Sladek J., Sladek V. (2017). Three-dimensional analysis for functionally graded piezoelectric semiconductors. J. Intell. Mater. Syst. Strut..

[18] Zhao M.H., Li Y., Yan Y., Fan C.Y. (2016). Singularity analysis of planar cracks in three-dimensional piezoelectric semiconductors via extended displacement discontinuity boundary integral equation method. Eng. Anal. Bound. Elem..

[19] Fan C.Y., Yan Y., Xu G.T., Zhao M.H. (2016). Piezoelectric-conductor iterative method for analysis of cracks in piezoelectric semiconductors via the finite element method. Eng. Fract. Mech..

[20] Zhang Q.Y., Fan C.Y., Xu G.T., Zhao M.H. (2017). Iterative boundary element method for crack analysis of two-dimensional piezoelectric semiconductor. Eng. Anal. Bound. Elem..

[21] Zhao Y.F., Zhou C.G., Zhao M.H., Pan E., Fan C.Y. (2017). Penny-shaped cracks in three-dimensional piezoelectric semiconductors via Green’s functions of extended displacement discontinuity. J. Intell. Mater. Syst. Strut..

[22] Danzer R. (2014). On the relationship between ceramic strength and the requirements for mechanical design. J. Eur. Ceram. Soc..

[23] Jelitto H., Kessler H., Schneider G.A., Balke H. (2005). Fracture behavior of poled piezoelectric PZT under mechanical and electrical loads. J. Eur. Ceram. Soc..

[24] Sato N., Takahashi K. (2018). Evaluation of Fracture Strength of Ceramics Containing Small Surface Defects Introduced by Focused Ion Beam. Materials.

[25] Ancona M.G., Binari S.C., Meyer D.J. (2012). Fully coupled thermoelectromechanical analysis of GaN high electron mobility transistor degradation. J. Appl. Phys..

[26] De S.C., Meneghini M., Caria A., Dogmus E., Zegaoui M., Medjdoub F., Kalinic B., Cesca T., Meneghesso G., Zanoni E. (2018). GaN-Based Laser Wireless Power Transfer System. Materials.

[27] Zhang D.L., Cheng X.H., Zheng L., Shen L.Y., Wang Q., Gu Z.Y., Qian R., Wu D.P., Zhou W., Cao D. (2018). Effects of polycrystalline AlN filmon the dynamic performance of AlGaN/GaN high electron mobility transistors. Mater. Des..

[28] Latorre-Rey A.D., Sabatti F.F.M., Albrecht J.D., Saraniti M. (2017). Hot electron generation under large-signal radio frequency operation of GaN high-electron-mobility transistors. Appl. Phys. Lett..

[29] International Organization for Standardization (2003). Fine Ceramics (Advanced Ceramics, Advanced Technical Ceramics)—Test Method for Fracture Toughness of Monolithic Ceramics at Room Temperature by Single Edge Precracked Beam Method.

[30] Carlton H.D., Elmer J.W., Freeman D.C., Schaefferet R.D., Derkach O., Gallegos G.F. (2016). Laser notching ceramics for reliable fracture toughness testing. J. Eur. Ceram. Soc..

[31] Araneo R., Falconi C. (2013). Lateral bending of tapered piezosemiconductive nanostructures for ultra-sensitive mechanical force to voltage conversion. Nanotechnology.

[32] Pierret R.F. (1996). Semiconductor Device Fundamentals.

[33] Wang Z.L. (2012). Progress in piezotronics and piezo-phototronics. Adv. Mater..

[34] Araneo R., Bini F., Pea M., Notargiacomo A., Rinaldi A., Lovat G., Celozzi S. (2014). Current–voltage characteristics of ZnO nanowires under uniaxial loading. IEEE Trans. Nanotechnol..

[35] Sze S., Ng K. (2006). Physics of Semiconductor Devices.

[36] Khusayfana N.M., Qasrawib A.F., Khanfar K.H. (2017). Impact of Yb, In, Ag and Au thin film substrates on the crystalline nature, Schottky barrier formation and microwave trapping properties of Bi_2_O_3_ films. Mater. Sci. Semicon. Proc..

[37] Zhou L. (2002). Development and Characterization of Ohmic and Schottky Contacts for GaN and AlGaN Devices. Ph.D. Thesis.

[38] Yang J.S. (2005). An Introduction to the Theory of Piezoelectricity.

[39] Zhao M.H., Ma S.J., Lu C., Fan C.Y., Qin G.S. (2018). Influence of polarization on the electromechanical properties of GaN piezoelectric semiconductive ceramics. Ceram. Int..

[40] Vasileska D., Klimeck G. (2010). Computational Electronics: Semiclassical and Quantum Device Modeling and Simulation.

[41] Fang F., Yang W. (2000). Poling-enhanced fracture resistance of lead zirconate titanate ferroelectric ceramics. Mater. Lett..

[42] Lu C., Danzer R., Fischer F.D. (2002). Fracture statistics of brittle materials: Weibull or normal distribution. Phys. Rev. E.

[43] Fu R., Zhang T.Y. (2010). Effects of an electric field on the fracture toughness of poled lead zirconate titanate ceramics. J. Am. Ceram. Soc..

[44] Zhang T.Y., Zhao M.H., Liu G.N. (2004). Failure behavior and failure criterion of conductive cracks (deep notches) in piezoelectric ceramics I—The charge-free zone model. Acta Mater..

[45] Zhang T.Y., Liu G.N., Wang T.H., Tong P. (2007). Application of the concepts of fracture mechanics to the failure of conductive cracks in piezoelectric ceramic. Eng. Fract. Mech..

